# Proliferation-associated POU4F2/Brn-3b transcription factor expression is regulated by oestrogen through ERα and growth factors via MAPK pathway

**DOI:** 10.1186/bcr2809

**Published:** 2011-01-17

**Authors:** Samir Ounzain, Samantha Bowen, Chandrakant Patel, Rieko Fujita, Richard J Heads, Vishwanie S Budhram-Mahadeo

**Affiliations:** 1 Medical Molecular Biology Unit, UCL Institute of Child Health, 30 Guilford Street, London WC1N 1EH, UK; 2 Cardiovascular Division, King's College London, Department of Cardiology, The Rayne Institute, St Thomas's Hospital, Lambeth Palace Road, London SE1 7EH, UK

## Abstract

****Introduction**:**

In cancer cells, elevated transcription factor-related Brn-3a regulator isolated from brain cDNA (Brn-3b) transcription factor enhances proliferation *in vitro *and increases tumour growth *in vivo *whilst conferring drug resistance and migratory potential, whereas reducing Brn-3b slows growth both *in vitro *and *in vivo*. Brn-3b regulates distinct groups of key target genes that control cell growth and behaviour. Brn-3b is elevated in >65% of breast cancer biopsies, but mechanisms controlling its expression in these cells are not known.

**Methods:**

Bioinformatics analysis was used to identify the regulatory promoter region and map transcription start site as well as transcription factor binding sites. Polymerase chain reaction (PCR) cloning was used to generate promoter constructs for reporter assays. Chromatin immunoprecipitation and site-directed mutagenesis were used to confirm the transcription start site and autoregulation. MCF-7 and Cos-7 breast cancer cells were used. Cells grown in culture were transfected with Brn-3b promoter and treated with growth factors or estradiol to test for effects on promoter activity. Quantitative reverse transcriptase PCR assays and immunoblotting were used to confirm changes in gene and protein expression.

**Results:**

We cloned the Brn-3b promoter, mapped the transcription start site and showed stimulation by estradiol and growth factors, nerve growth factor and epidermal growth factor, which are implicated in breast cancer initiation and/or progression. The effects of growth factors are mediated through the mitogen-activated protein kinase pathway, whereas hormone effects act via oestrogen receptor α (ERα). Brn-3b also autoregulates its expression and cooperates with ERα to further enhance levels.

**Conclusions:**

Key regulators of growth in cancer cells, for example, oestrogens and growth factors, can stimulate Brn-3b expression, and autoregulation also contributes to increasing Brn-3b in breast cancers. Since increasing Brn-3b profoundly enhances growth in these cells, understanding how Brn-3b is increased in breast cancers will help to identify strategies for reducing its expression and thus its effects on target genes, thereby reversing its effects in breast cancer cells.

## Introduction

The class 4 POU (Pit-Oct-Unc) transcription factor-2- related to Brn-3 (POU4F2/Brn-3b), is referred to as Brn-3b because of homology in the DNA binding domain to the related Brn-3a transcription factor. Brn-3b is highly expressed in a significant proportion (> 65%) of breast tumour biopsies analyzed [1]. Over-expression of Brn-3b in cancer cells is strongly associated with increased proliferation,* in vitro*, and enhanced tumour growth, *in-vivo*, whereas reducing Brn-3b (by antisense) decreases proliferation *in-vitro *and results in smaller, slower growing tumours *in-vivo *[2,3]. Brn-3b also confers resistance to growth inhibitory or apoptosis inducing chemotherapeutic drugs but also increases migratory potential of cancer cells [2]. Recent studies also showed that Brn3b is increased in doxorubicin resistant breast cancer cells (R Fujita and V Budhram-Mahadeo, unpublished data).

As a transcription factor, Brn-3b regulates the expression of critical genes that control different cellular processes. For example, increased proliferation by Brn-3b may be associated with its ability to transactivate the promoters of genes required for cell cycle progression such as cyclin-dependent kinase 4 (*CDK4*) [4] and its regulatory partner *cyclin D1 *[5], which are required, whilst repressing breast cancer susceptibility gene 1 (*BRCA1*) [6], which is associated with cell cycle arrest in breast cancer cells. Invasiveness and drug resistance associated with Brn-3b in cancer cells are linked with its ability to transactivate genes such as the small heat shock protein 27 (*HSP27*) [7] whilst repressing promoters of genes encoding adhesion molecules, for example, *γ-catenin/plakoglobin *[8].

However, whilst the effects of increased Brn-3b in cancer cells have been characterised and many of its target genes have been studied, we do not know which factors contribute to the elevated *Brn-3b *mRNA and protein levels observed in breast cancer. In this study, we have cloned and analysed the regulatory region that controls *Brn-3b *gene expression in MCF-7 breast cancer cells. The results presented herein identify a proximal promoter present in the 5' sequences upstream of the *Brn-3b *gene which drives expression in MCF-7 cells. This promoter is transactivated by the growth factors nerve growth factor (NGF) and epidermal growth factor (EGF) and the hormone estradiol, all of which are known to promote the proliferation and/or survival of breast cancer cells. NGF and EGF increase promoter activity by signalling through the p42/p44-(ERK) mitogen-activated protein kinase (MAPK) pathway, whereas the effects of oestrogen are mediated via oestrogen receptor α (ERα) but not oestrogen receptor β (ERβ). We also show autoregulation by Brn-3b to increase its own expression. These findings suggest that increased transcription of Brn-3b in breast cancer cells is stimulated by growth factors and hormones that enhance proliferation and propagate through autoregulation.

## Materials and methods

### Materials

General laboratory reagents were purchased from Merck (Nottingham, UK) and Sigma (Dorset, UK) unless otherwise stated. Primary antibodies were used at dilutions of 1: 1000-1500 and included Brn-3b-rabbit pAb (Abcam-Cambridge, UK); Brn-3b-goat pAb (Santa Cruz Biotechnology Inc, USA); actin - goat pAb (I-19, Santa Cruz Biotechnology). HRP-conjugated secondary Ab from Dako (Cambridgeshire, UK) was used for immunoblotting 1:2000). Estradiol (E4389), cyclic adenosine monophosphate (cAMP), phorbol 12,13-dibutyrate (PDBu) and 4-hydroxytamoxifen (tamoxifen) were from Sigma (Dorset, UK); epidermal growth factor (EGF), transforming growth factor-β (TGF-β); insulin-like growth-1 (IGF 1) and nerve growth factor (NGF) were from Roche Diagnostics GmbH (Welwyn Garden City, Hertfordshire, UK). Signalling pathway inhibitors PD-98059 (MEK), SB-203580 kinase (p38), Genistein (tyrosine kinase), and Wortmannin (PI3-kinase) were from Calbiochem (Nottingham, UK). The MCF7 breast cancer cell line was obtained from ATCC. Expression vectors, Brn-3b(l); Brn-3b(s); ER were previously described [9]. Dominant negative and constitutively active MEK expression vectors were kind gift from D.S. Latchman[10].

### *In silico *analysis of Brn-3b promoter

*Homo sapiens *chromosome 4 contig was analysed using the Basic Local Alignment Search Tool, or BLAST [11], to identify a region containing the *Brn-3b *gene consisting of approximately 10 kb sequence (in bacterial artificial chromosome clone AC093887). Further analysis using Bioinformatics and Molecular Analysis Section (BIMAS) ProScan software [12] was used to identify putative promoter sequences in this region of DNA. The VISTA Genome Browser [11] was used to generate homology plots, whereas analysis using Genomatix TRANSFAC software analysis (Genomatix, Munich, Germany) identified binding sites for transcription factors in the putative promoter sequences.

### Brn-3b reporter constructs

**Figure 1:**
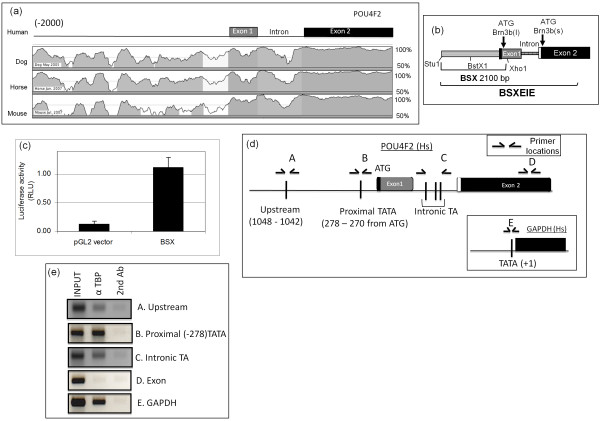
**Identification and cloning of transcription start site in transcription factor-related Brn-3a regulator isolated from brain cDNA (Brn-3b) promoter**. **(a) **Homology plots (VISTA Genome Browser) showing regions of similarity in *Brn-3b *gene and 5' upstream sequences between human (top) and dog, horse and mouse (bottom) genome sequences. Regions of homology are indicated by peaks and grey shading, and positions of Brn-3b exons and intronic sequences are shown. **(b) **Schematic showing cloned *Bst*X1 (B)/*Stu*1 (S)/*Xho*1 (X) (BSX) construct containing putative Brn-3b promoter and regulatory sequences or the BSX exon-intron-exon (BSXEIE) expression construct containing the promoter, regulatory and coding sequences, which were used for subsequent studies. Promoter and regulatory sequences are shown in grey-striped area. 5' noncoding sequences at the beginning of exon 1 are indicated by the black bar. The white stripe at the start of exon 2 represents unique sequences that are present in Brn-3b(s) transcripts, but not in Brn-3b(l) transcripts. The positions of restriction enzyme sites *Bst*X1 (B), *Stu*1 (S) and *Xho*1 (X) used for cloning are also shown. **(c) **Luciferase activity of Brn-3b reporter constructs following transfection into MCF-7 cells is compared with baseline luciferase activity of the empty reporter vector. Values, shown as relative luciferase units (RLU),, were equalised with Renilla internal control **(d) **Schematic showing positions of putative start sites identified by *in silico *analysis. Initiator element and proximal TATA sequences are shown relative to ATG in exon 1, and putative intronic TATA sequences are indicated. Half-arrows show relative positions of primers used for polymerase chain reaction (PCR) assay following chromatin immunoprecipitation (ChIP) assay with α-TATA box binding protein (α-TBP) antibody (Ab) to analyse TBP binding to the different sites. **(e) **PCR products obtained when ChIP DNA (obtained with α-TBP Ab or secondary control Ab) was used for amplification with primers that flanked the upstream initiator elements (-1,048 bp) (A), -278TATA (B) or the intronic TA sequences (C). Primers for sequences within exon 2 (> 1 kb from ATG) were used for amplification in the negative control (D). Positive controls represent PCR products derived by using primers that amplified the known start site of the glyceraldehyde 3-phosphate dehydrogenase (*GAPDH*) gene using α-TBP or control ChIP DNA (E). Input represents amplification using one-tenth of DNA isolated before performing the ChIP assay. Right column (labeled 2nd Ab) shows the products obtained following amplification of control ChIP Ab (α-rabbit secondary Ab) using the indicated primers.

Brn-3b reporter constructs were generated so that the regulatory promoter region drove expression of a firefly luciferase reporter gene in the pGL2 plasmid. The initial Brn-3b reporter construct was generated by amplifying ~1,400 bp regions upstream of the *Brn-3b *gene sequence and incorporating part of exon 1 (Figure 1b). The resultant construct was designated *Bst*X1/*Stu*1/*Xho*1 (BSX) because it included sequences that can be isolated using restriction *Bst*X1, *Xho*1 site (exon 1) and *Stu*1 site and were used for diagnostic digestion. The BSX exon-intron-exon (BSXEIE) construct was subsequently generated by cloning the gene encoding sequence (exon 1, intron and exon 2) upstream of this putative regulatory region, thus allowing Brn-3b promoter to drive its own gene expression.

### Chromatin immunoprecipitation assay

The chromatin immunoprecipitation (ChIP) assay was carried out as described by Lee *et al*. [7]. In studies to identify the transcription start sites, anti-TATA box binding protein (anti-TBP) Ab (Abcam) was used to immunoprecipitate regions of promoter bound by TBP in the transcription initiation complex. Later studies to confirm Brn-3b binding in its own promoter was done using antigoat Brn-3b Ab (Santa Cruz Biotechnology) to immunoprecipitate Brn-3b bound to chromatin in intact cells. Negative control ChIP assay was performed using antibody to glyceraldehyde 3-phosphate dehydrogenase (anti-GAPDH) (Abcam) or secondary Ab (Dako) only. The shear size of DNA following ChIP assay and sonication was ~200 to 600 bp as determined for agarose gel electrophoresis. The PCR assay for the transcriptional start site was performed on ChIP DNA using primers designed to amplify different regions of the putative Brn-3b promoter as follows: Upstream initiator: forward 5'-CTTGGGCCGCAACTTTATT-3' and reverse 5'-TACCTAAGGACCAGCCTCCA-3'; -278TATA: forward 5'-CGGGGAGAGGGGAGTATAAC-3' and reverse 5'-GATGCCTGACTCCGCTTG-3'; intronic TA: forward 5'-TTGACAGCCCCCTTTATCTG-3' and reverse 5'-AGGCAACATCCCAGGTCATA-3'; and negative control primers which amplified the exon 2 sequence: forward 5'-ACCATGAACCCCATGCAC-3' and reverse 5'-CTTGATGCCTCGCTGCTT-3'. The distance between the intronic site and the exonic sequences amplified was ~1 kb. As a positive control, the following primers were used to amplify the GAPDH promoter start site: forward 5'-TGAGCAGACCGGTGTCAC-3' and reverse 5'-AGGACTTTGGGAACGACTGA-3'. Primers used to amplify the promoter region containing the Brn-3b site were as follows: forward 5'-GCCCCTTCTTCCTTTGATTG-3' and reverse 5'-ACACACACACGCTCCTCTTG-3'. Standard conditions for PCR amplification included 2.5 mM MgCl_2 _and the following cycling parameters: 1 cycle at 94°C for 15 minutes followed by 40 cycles of amplification for each experiments using 95°C for 30 seconds, at 58°C for 30 seconds, and at 72°C for 30 seconds. A final cycle was undertaken at 72°C for 5 minutes, the complete elongation steps and the PCR products were then resolved on a 2.5% agarose/Tris-borate-ethylenediaminetetraacetic acid gel.

### Site-directed mutagenesis

Site-directed mutagenesis was carried out to test the effects of altering alter key bases in either the different putative transcriptional start sites or transcription factor binding sites, such as Brn-3b site or oestrogen response element (ERE), in the Brn-3b promoter. This was achieved using the QuickChange Site-Directed Mutagenesis Kit (Stratagene, Stockport, Cheshire, UK) and experiments were carried out in accordance with the manufacturer protocol. Primers used to mutate the Brn-3 site (located at -1,324 bp to -1,312 bp from ATG) were forward 5'-CCCTTCTTCCTTTGATTGTGGCTAATGAAGAAGGATCCATCCAGGGG CAGGGTTT-3' and reverse 5' AAACCCTGCCCCTGGATGGATCCTTCTTCATTAGC CACAATCAAAGGAAGAAGGG-3'. Primers used to mutate ERE (located at -1,263 bp to -1,255 bp from ATG) were forward 5'-CATATGCGCTGTGTAATTT CTGGAATTCCCTC TCCCTGTCAGTTG-3' and reverse 5'-CAACTGACAGGGAGAGGGAATTCCAGAAAT TACACAGCGCATATG-3'. Primers used to alter the upstream initiator (located at -1,048 bp to -1,042 bp) were forward 5' CCAACGCTGGCTTGGGCCGCAACTCTAGATGGGAGTTTT CTTTTTC-3' and reverse 5'-GAAAAAGAAACTCCCATCTAGAGTTGCGGCCCAAG CCAGCGTTGG-3'. To mutate the proximal TATA (located at -278 bp to -271 bp from ATG), we used the following primers: forward 5'-GCGGGGAGAGGGGGGTACCCCTCGCCGG CCGCG-3', reverse 5'-CGCGGCCGGCGAGGGGTACCCCCCTCTCCCCGC-3'. To mutate intronic TATA (located at +293bp to +297 bp from ATG), we used the following primers: forward 5'-GTCTTCCAACCCCACCGGTGGGTACCCCTGCATAATCACCGCTTA AA-3' and reverse 5'-TTTAAGCGGTGATTATGCAGGGGTACCCACCGGTGGGGTTGGAAGAC-3'. Consecutive rounds of mutagenesis were performed to generate double or triple mutants. Restriction analysis, together with DNA sequencing, confirmed the resulting mutations.

### Western blot analysis

Total cellular protein preparation and immunoblotting were undertaken as previously described [6] with 1 hour block in phosphate-buffered saline Tween-20, primary Ab incubation for 1 to 3 hours and secondary Ab incubation for 45 to 60 minutes. Signals were developed using enhanced chemiluminescence reagent (Amersham, Buckinghamshire, UK).

### Cell culture, transient transfections and reporter assays

MCF-7 breast cancer cells were maintained in Dulbecco's modified Eagle's medium (DMEM) supplemented with 10% fetal calf serum, 1% nonessential amino acids and 1% penicillin/streptomycin. Cells were plated onto six-well plates (5 × 10^4 ^cells/well) 24 hours before transfection with reporter and expression vectors using FuGENE HD Transfection Reagent (Roche, Welwyn Garden City, Hertfordshire, UK) or GeneJuice Transfection Reagent (Merck Biosciences, Nottingham, UK). Transfection was undertaken according to the manufacturer's protocol. To reduce the activity of endogenous ER, cells were grown in oestrogen-depleted medium, that is, phenol red minus DMEM supplemented with charcoal-stripped FCS [9], for up to 72 hours before transfection and subsequent analysis. Forty-eight hours following transfection promoter activity was measured using the Dual-Luciferase Reporter Assay System (Promega, Southampton, UK) according to the manufacturer's protocol using a TD 20/20 luminometer (Turner Designs, Promega, Southampton, UK). Internal control Renilla luciferase reporter activity was used to control for variations in transfection efficiency, and values are expressed as percentages of empty vector control.

## Results

### Identification of the Brn-3b promoter

Bioinformatics analysis of 5' sequences upstream of the Brn-3b coding sequence using the VISTA Genome Browser [13,14] revealed regions of high conservation across different species (Figure 1a). Such sequence homology often indicates key functions [12], so *in silico *analysis was undertaken for regulatory sequences in this noncoding region. Using BIMAS ProScan software [12], we identified putative transcription initiation sequences (for example, TATA or initiator elements) within the proximal sequences (Figure 1a), which can be indicative of promoters. Furthermore, analysis of the sequence using MatInspector Transcription Factor Analysis Tool software (TransFac, Genomatix) led to the identification of putative binding sites for transcription factors that are known to regulate the growth of cancer cells, for example, estrogen receptor element (ERE), epidermal growth factor response element (EGRF) and serum response element (SRE). Because of the high conservation across species, we examined whether polymorphism in these sequences might contribute to elevated Brn-3b expression in breast cancer biopsies by sequencing and comparing genomic DNA from 15 primary breast biopsies (including normal breast and breast cancer biopsies) with the breast cancer cell lines HB4a and MCF-7. No significant polymorphisms were observed, except in microsatellite sequences (data not shown), suggesting that the increased Brn-3b mRNA observed in breast tumours might result from activation of its promoter by upstream growth effectors and/or signalling pathways that stimulate gene transcription.

### Cloning of promoter and mapping transcription start site

To identify factors that stimulate Brn-3b promoter activity and therefore gene expression in breast cancer cells, the BSX reporter construct, containing the putative Brn-3b promoter and regulatory sequences cloned into pGL2 basic reporter vector (see Materials and methods, and Figure 1b) was used in transfection studies. Figure 1c shows high basal activity from the Brn-3b promoter construct compared with empty pGL empty vector control, thereby confirming that these sequences were sufficient to promote reporter gene expression. The BSXEIE construct containing additional sequences, including the intron region, give rise to similar results (not shown).

To identify sites from which transcription may be initiated on this promoter, an *in vivo *ChIP assay was undertaken using an antibody to the TBP component of the basal transcriptional complex [13]. Primers were designed to amplify regions that flanked putative transcription start sites, as shown in Figure 1d (position in reference to first ATG), and referred to as upstream initiator sequence (at position -1048 to -1042) or proximal TATA-like sequence (located at -278 to -272). The primers used to amplify an intronic region with TA-like elements were also tested because this region was found to have an alternative promoter in the related *Brn-3a *gene, which has a genomic arrangement similar to that of *Brn-3b*. The primers for sequences in exon 2 were used as negative controls.

Figure 1e shows the PCR products obtained following amplification of α-TBP ChIP DNA using primers for different putative start sites in the promoter. Figure 1e (lane B) shows that primers flanking the putative proximal TATA site at -278 (-278TATA) produced a strong band that was not seen when these primers were used to amplify control ChIP DNA (secondary Ab). This product was comparable to the positive control PCR product obtained using primers that amplified the known start site in the *GAPDH *gene (Figure 1e, lane E), suggesting significant TBP binding to this proximal TATA-containing region of the promoter. In contrast, amplification of sequences spanning the putative upstream initiator element (Figure 1e, lane A) or intronic regions (Figure 1e, lane C) gave rise to faint bands. This may result either from weak binding of TBP to these regions or from variability in shear size of ChIP DNA. No bands were seen with primers amplifying exon 2 (negative control) (Figure 1e, lane D), indicating the specificity of the assay. The data therefore suggest significant binding of TBP to proximal TATA and possibly weak binding to initiator elements and sequences within the intron.

**Figure 2:**
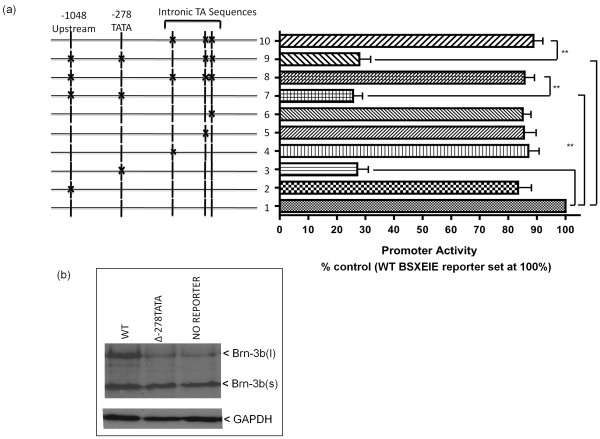
**Analysing the transcription start sites of Brn-3b promoter**. **(a) **Analysis of Brn-3b promoter (BSX) activity following the mutation of key sites to identify the transcriptional start site. Wild-type (WT) promoter in row 1 (right) is represented as 100%, and all mutations are expressed as percentages of the WT promoter. Schematic shows positions of mutations at key sites (indicated by x, left), for example, upstream initiator alone (row 2, right) or in combination with other sites (rows 7 to 9, right). The proximal -278TATA mutation is shown alone (row 3, right) or in combination (rows 7 and 9, right). The effects of mutation of different intronic TA alone (rows 4 to 6, right) or in combination (rows 8 to 10, right) are also shown. Values are equalised to internal control (Renilla luciferase), and the results represent data from three independent experiments expressed as means + SD. * is used to show statistical significance between different mutant promoter constructs and wild-type constructs. **(b) **Western blot analysis of Brn-3b protein expression in cellular extracts prepared from MCF7 cells transfected with BSXE1E expression constructs in which Brn-3b promoter drives expression of the Brn-3b gene. WT promoter activity is shown in left column. Middle column (Δ-278TATA shows the reduction in Brn-3b protein from expression constructs containing a mutation within the proximal -278TATA promoter of an otherwise, intact construct. Right column shows untransfected control cells with no reporter and therefore represents levels of endogenous Brn-3b protein in these cells.

To confirm which of these sites was required for transcription initiation, site-directed mutagenesis was used to alter bases at the proximal -278TATA site, the upstream site (-1,042) or within the intronic TA sequences either alone or in different combinations (Figure 2a). Mutated constructs were used for similar transfection assays, and the results, shown in Figure 2b, demonstrate that mutation of -278TATA alone (Δ-278TATA column) resulted in significantly reduced promoter activity (by 70% to 80%) compared with the WT promoter (set at 100% - Figure 2a, right, row 1). Furthermore, when proximal -278TATA was mutated in any combination (with initiator-like elements at -1,042 or intronic TA), a similar loss of promoter activity was observed (Figure 2a, right, rows 7 and 9). However, mutation of upstream initiator-like elements (-1,048 to -1,042) alone (Figure 2a, right, row 2) or intronic TATA-like elements alone or in combination (Figure 2a, right, rows 4 to 6) did not reduce promoter activity if -278TATA was intact. These results suggest that the proximal TATA element (-278 from ATG) is essential for the formation of basal promoter complex required to drive expression from the Brn-3b promoter and hence will mark the vicinity of the transcriptional start site. The intronic TA and distal initiator element did not appear to be sufficient or required for transcriptional initiation, independently of this proximal TATA, in breast cancer cells.

Since -278TATA is necessary for transcriptional activity, we next tested whether altering this element was sufficient to reduce Brn-3b protein expression in these cells. For the studies, we used the BSXEIE constructs, in which the WT or mutant Brn-3b promoter (in which residues in -278TATA were altered in the otherwise intact promoter) was cloned upstream of its own coding sequence (comprising EIE) and therefore drives its own expression. Following transfection, protein extracts from cells transfected with WT or mutated -278TATA were used for immunoblotting to measure exogenous Brn-3b protein produced from the transfected BSXEIE construct compared with baseline expression (in empty vector transfected cells). Figure 2b (WT column) shows increased Brn-3b protein levels in cells expressing the WT construct (with intact -278TATA driving *Brn-3b *gene expression) compared with basal levels in untransfected control cells ("No Reporter" column). This was more evident for the longer Brn-3b(l) isoform because basal levels expressed in control cells are much lower compared with the shorter Brn-3b(s) isoform. However, mutation of -278TATA (in the otherwise intact construct) resulted in loss of this induction of Brn-3b protein (Δ-278TATA column) since levels were similar to endogenous expression in control cells. On the basis of the results of these different studies, we concluded that the proximal TATA located at position -278 from ATG (-278TATA) marks the transcription start site for Brn-3b transcription breast cancer cells.

### Brn-3b promoter is stimulated by NGF and EGF via the MAPK pathway

**Figure 3:**
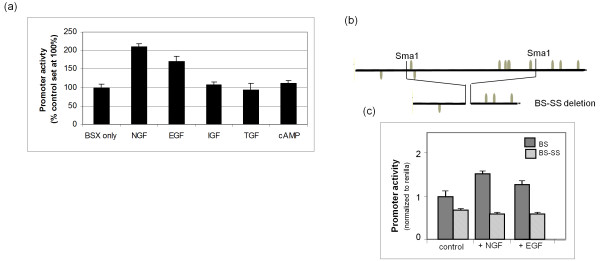
**Analysing the effect of growth factors on Brn-3b promoter activity**. **(a) **Brn-3b promoter (BSX) activity was measured following transfection of the reporter construct into MCF-7 cells and treatment with different growth factors. Values were equalised with internal control and Renilla luciferase activity and expressed as a percentage of promoter only (set at 100%). The data shown represent means ± SD from at least three independent experiments. NGF, nerve growth factor; EGF, epidermal growth factor; IGF, insulin-like growth factor; TGF, transforming growth factor; cAMP, cyclic adenosine monophosphate. **(b) **Schematic showing the position of growth factor response element (EGRF) and serum response element (SRE) sites in the Brn-3b promoter. The location of two *Sma*1 restriction enzyme sites, which were used to generate the deletion (*Sma1/Sma1*) construct, BS-SS, is shown in relation to the DNA binding sites. The resultant truncated promoter is represented schematically below. **(c) **Luciferase activity of intact (BS) promoter (control) or *Sma*1/*Sma1 *deletion promoter (BS-SS) is shown following transfection into MCF-7 cells. Grey bars represent WT promoter activity either alone or following treatment with NGF or EGF, whereas stippled bars show the activity of the *Sma*1/*Sma*1 deletion construct with or without growth factors. Values represent means ± SE of three independent experiments.

Since Brn-3b mRNA is increased in breast cancers, we next tested whether this promoter is regulated by growth factors that alter proliferation of these cancer cells. Therefore, MCF7 cells, transfected with the BSX promoter, were treated with known growth regulators including cyclic AMP (cAMP); epidermal growth factor (EGF); nerve growth factor (NGF) and insulin-like growth factors (IGF-1) [14-17]. Transforming growth factor (TGFβ), which is an inhibitor of cell growth[18], was also tested. Figure 3a shows stimulation of Brn-3b promoter activity by NGF (300 ng/ml) and EGF (50 ng/ml) whereas IGF-I (1-100 nM), TGFβ (0.5-20 ng/ml) and cyclic AMP (1 uM) had no effect on its activity in these cells. Both NGF and EGF could stimulate this promoter at a range of different concentrations tested (100-500 ng/ml for NGF and 1-100 ng/ml for EGF) (data not shown).

Analysis of the Brn-3b promoter using MatInspector TransFac Analysis Tool software identified multiple transcription factor binding sites for transcription factors stimulated by these growth factors, for example, EGR and NGF induced protein C. Therefore, we tested whether this region of the promoter was necessary for promoter stimulation by specific growth factors. Because of the presence of multiple sites in this region of the promoter, it was necessary to generate deletion constructs instead of mutating individual sites. Therefore, *Sma*1 restriction enzyme sites were used to delete a region of the promoter containing six EGFR and SRE sites by restriction enzyme digestion and religation. The resultant deletion promoter construct generated following *Sma1*/*Sma1 *digests (SS), which was designated BS-SS (shown in Figure 3b), was used in similar cotransfection assays, with or without NGF or EGF. Figure 3c shows that the BS-SS deletion reporter construct was no longer stimulated by NGF or EGF, as seen in the WT promoter. Although basal activity was slightly lower than that of the WT promoter, this did not account for the loss of inducibility by NGF and EGF, suggesting that key DNA binding sites present in this region are essential for increasing promoter activity in breast cancer cells.

**Figure 4:**
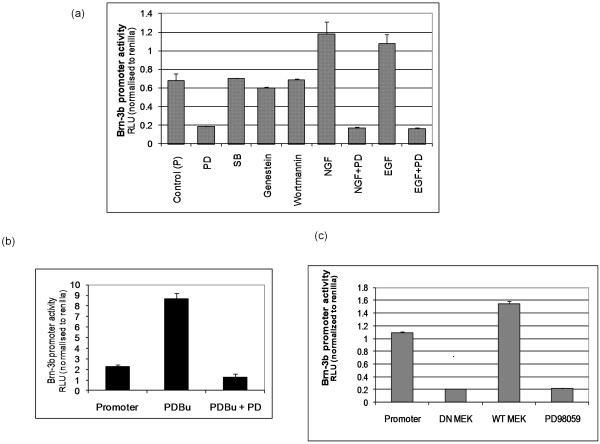
**Brn-3b promoter is activated via the mitogen-activated protein kinase/extracellular signal-regulated kinase (MAPK/ERK) pathway in breast cancer cells**. **(a) **Brn-3b promoter activity is shown following treatment of transfected cells with inhibitors of different signalling pathways (as indicated), in absence or presence of NGF or EGF. Values have been adjusted using internal Renilla luciferase control and represent means ± SD of at least three independent experiments. **(b) **Increase in Brn-3b promoter activity following treatment of transfected cells with the protein kinase C analogue phorbol 12,13-dibutyrate in the absence or presence of the ERK inhibitor, PD98059. Values represent relative luciferase activity after adjusting for Renilla internal control. **(c) **Brn-3b promoter activity is shown following cotransfection with either dominant-negative mitogen-activated protein kinase kinase (dnMEK) or WT MEK compared with activity in the presence of the ERK inhibitor PD98059. Values represent relative luciferase activity after adjusting for Renilla internal control.

NGF and EGF act as ligands, which, when bound to specific receptors, activate signalling pathways that alter downstream transcription factors, which in turn modulate downstream gene expression [16,19]. To identify pathways that modify promoter activity, cells transfected with the Brn-3b reporter construct were treated with pharmacological inhibitors or activators of key signalling pathways. Figure 4a shows that PD98059, an inhibitor of the p42/p44 MAPK pathway, strongly and specifically repressed endogenous Brn-3b promoter activity, whereas inhibitors of other pathways, for example, SB203580 (p38 kinase inhibitor), Genistein (tyrosine kinase inhibitor) or Wortmannin (PI3K inhibitor), had no effect on promoter activity. Furthermore, PD98059 blocked activation by NGF and EGF, suggesting that these growth factors stimulate Brn-3b promoter activity by signalling through the p42/p44 MAPK pathway. Interestingly, strong induction of promoter activity by PDBu, a potent activator of PKC (Figure 4b) was also inhibited by PD98059, suggesting an important role for the p42/p44 MAPK signalling pathway in controlling Brn-3b promoter activity in breast cancer cells through different upstream activators.

To confirm the requirement for the p42/p44 MAPK pathway in stimulating this promoter, we overexpressed WT MEK1 (an upstream activator of p42/p44 MAPK) or dnMEK1 with the Brn-3b reporter construct using cotransfection protocols. Figure 4c shows that increasing WT MEK1 could stimulate endogenous promoter activity, whereas the dnMEK1 construct reduced basal promoter activity to levels seen with PD98059 treatment. Thus, Brn-3b promoter activity can be inhibited by blocking the MAPK/extracellular signal-regulated kinase (ERK) pathway by using either pharmacological inhibitors or dnMEK, thereby identifying the MAPK/ERK pathway as a pivotal regulator of Brn-3b expression in breast cancer cells.

### Activation of Brn-3b promoter by the hormone 17β-estradiol occurs via ERα but not ERβ

**Figure 5:**
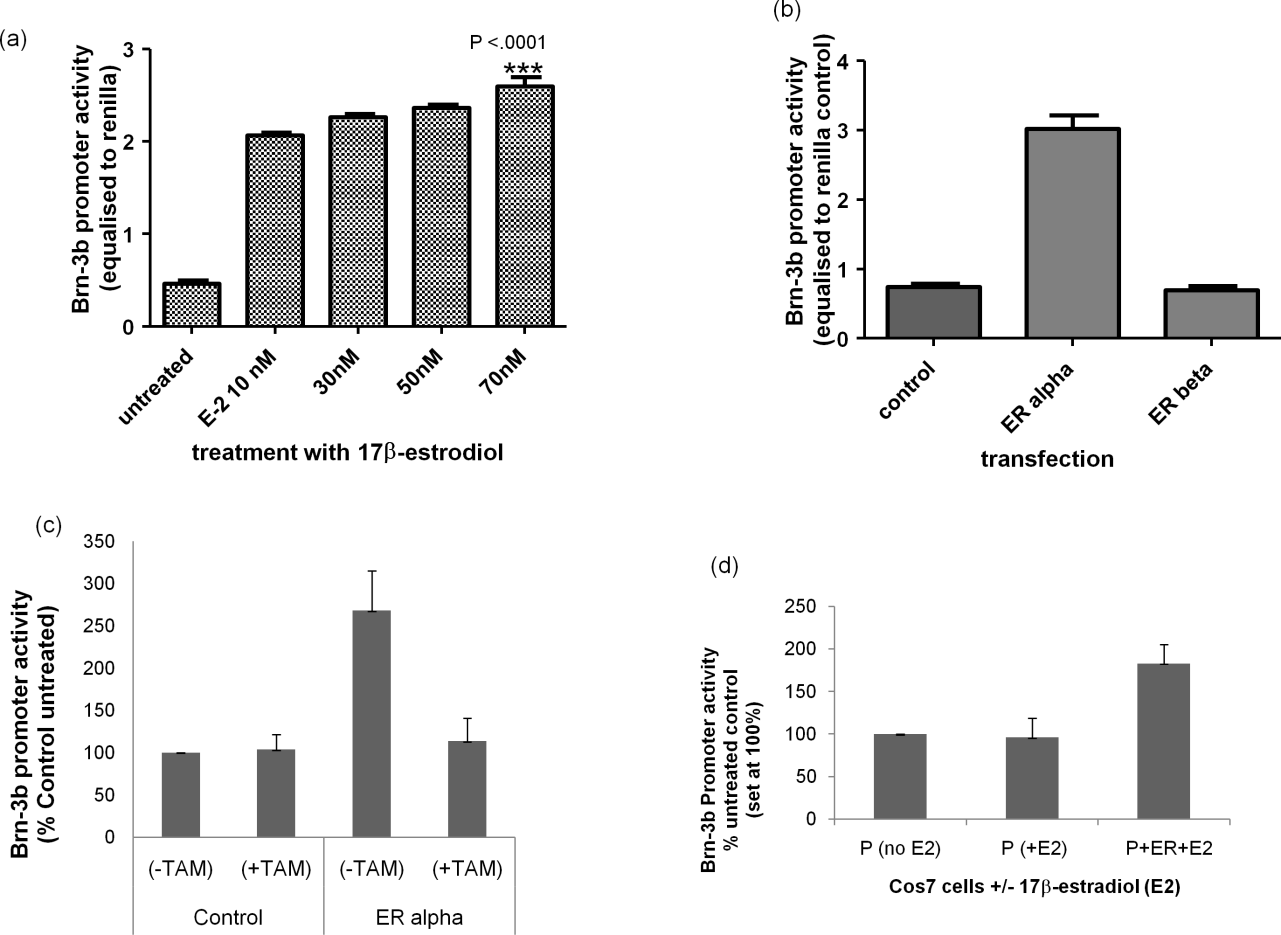
**Brn-3b promoter activity is strongly stimulated by 17β-estradiol via activation of oestrogen receptor α (ERα)**. **(a) **Brn-3b promoter activity following treatment of transfected MCF-7 cells with different concentration of 17β-estradiol. Values have been adjusted with internal control, Renilla luciferase and represent means ± SD of three independent experiments ***indicate statistical significance of p < 0.0001 compared with control. **(b) **Effect of different oestrogen receptors, ERα or ERβ on Brn-3b (BSX) promoter activity are shown after cotransfection into sensitised MCF-7 cells (grown in stripped serum medium for 48 hours). Promoter activity is adjusted to internal Renilla control and expressed as percentage of levels seen with empty vector only (set at 100%). Values represent data from three independent experiments, expressed as means ± SD. **(c) **Activation of Brn-3b promoter by ERα can be blocked by the receptor antagonist tamoxifen. Promoter activity is shown after cotransfection of Brn-3b promoter with ERα into sensitised MCF-7 cells (grown in oestrogen-depleted medium for 48 hours) in the presence of 1 μM 4 hydroxy tamoxifen (TAM) compared with untreated control. Promoter activity is adjusted to internal Renilla control and expressed as percentages of levels seen with untreated controls (set at 100%). **(d) **Brn-3b promoter activity in ER-negative Cos-7 cells treated with estradiol and either transfected ERα or control vector. Data shows luciferase activity in cells transfection of Brn-3b promoter and treatment with either vehicle (-E2) or 10 nM estradiol (+E2) in the absence or presence of ERα.

The hormone oestrogen plays a critical role in the initiation and progression of many breast cancers because breast epithelial cells are highly responsive to its proliferative effects. Therefore, we tested whether active oestrogen (17β-estradiol) could stimulate Brn-3b promoter activity using MCF-7 cells sensitized to estradiol by growth in stripped-serum, phenol-red less DMEM [9]. Cells transfected with the Brn-3b promoter construct were either untreated or treated with different concentrations of 17β-estradiol. Figure 5a shows that 17β-estradiol significantly increased promoter activity compared with untreated cells, suggesting that this hormone can stimulate Brn-3b transcription in breast cancer cells, thereby contributing to downstream oestrogenic growth effects.

Estradiol can act through one of two receptors: ERα or ERβ. Of these, increased ERα is implicated in the etiology of breast cancers and is often targeted for treatment. We therefore tested the effects of coexpressing either ERα or ERβ on Brn-3b promoter activity. Figure 5b shows that the promoter was strongly stimulated by ERα, whereas ERβ did not alter its activity, suggesting that the effects of oestrogen in breast cancer cells are likely to be mediated via ERα. As expected, the addition of the ER antagonist tamoxifen prevented activation of the Brn-3b promoter by oestrogen (Figure 5c), thus confirming that this receptor is required for stimulation of Brn-3b promoter activity in MCF-7 cells. This finding was further supported by studies carried out in ER-negative Cos-7 cells, which showed that estradiol did not activate the Brn-3b promoter unless exogenous ER was introduced following transfection (Figure 5d). These results suggest that ERα is necessary to mediate the effects of oestrogens in MCF-7 breast cancer cells but can also act independently of oestrogen to increase Brn-3b transcription.

### Autoregulation by Brn-3b and cooperation with ERα also increases promoter activity

**Figure 6:**
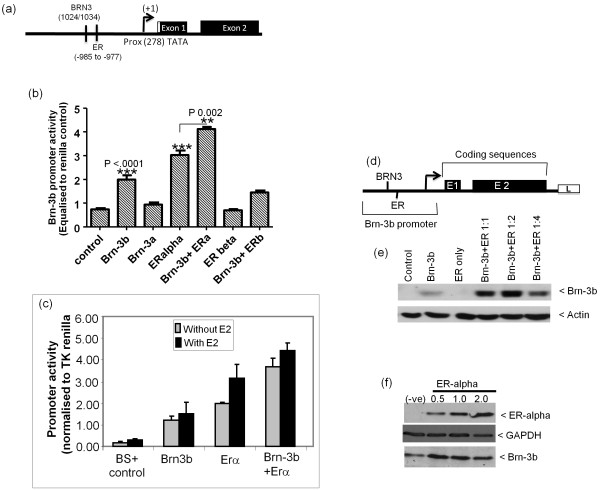
**Cooperation between Brn-3b and ER can stimulate promoter activity**. **(a) **Schematic diagram showing position of Brn-3 consensus binding sites and oestrogen response element (ERE) site relative to the proximal -278TATA, (now designated +1). **(b) **Promoter activity following cotransfection of the BSX reporter construct with Brn-3b, Brn-3a, ERα or ERβ alone or in different combinations into MCF-7 cells. Values have been equalised with internal Renilla control [renilla reporter gene driven by minimal tyrosine kinase (TK) promoter] and represent means ± SD of three independent experiments. **(c) **Brn-3b promoter activity when coexpressed with Brn-3b and ERα, alone or together, in cells grown in oestrogen-depleted medium and either untreated or treated with estradiol, after transfection. Grey bars show inducibility in the absence of estradiol, and black bars demonstrate the effects of adding estradiol after transfection with the constructs described. **(d) **Schematic showing the modified expression construct, BSXE1E, in which the Brn-3b promoter drives expression of the Brn-3b gene upstream of luciferase reporter (L). **(e) **Immunoblot showing changes in Brn-3b protein produced from the BSXE1E expression construct when cotransfected with Brn-3b or ERα alone or in combination (at different ratios of Brn-3b:ER, 1:1 to 1:4). Actin immunoblot was used to indicate variation in protein loading. To demonstrate that Brn-3b protein levels was dependent on the ratio of Brn-3b to ER, shorter exposure times and therefore smaller increases in Brn-3b expression are not evident when ER only is expressed. **(f) **Immunoblot to show increases in ER protein following transfection with expression constructs (top immunoblot) and a corresponding changes in endogenous Brn-3b protein that were dependent on concentrations of ERα (middle immunoblot). GAPDH immunoblot is included to show variation in protein loading.

TRANSFAC software analysis revealed binding sites for Brn-3 proteins, suggesting that Brn-3b and/or a related family member, Brn-3a, may also regulate promoter activity. A putative ERE site was identified within proximity to this site (Figure 6a), and since previous studies demonstrated physical interaction between Brn-3b and ERα that could stimulate transcription of ERE-containing target genes, we tested whether Brn-3b could regulate its own promoter activity and cooperate with ERα to increase its own expression.

Figure 6b shows that Brn-3b could weakly transactivate its own promoter, whereas the related Brn-3a protein had no effect on promoter activity in these cells. Although ERα alone stimulated promoter activity, coexpression of this receptor with Brn-3b resulted in more significant increases. ERβ did not affect promoter activity with or without Brn-3b, suggesting that a specific and unique cooperation occurs between ERα and Brn-3b to stimulate the Brn-3b promoter in breast cancer cells. Studies carried out in sensitised MCF7 cells grown in "phenol red less" DMEM, containing stripped serum, to deplete oestrogenic activity, shows that exogenous (transfected) ERα could to stimulate Brn-3b promoter in the absence or presence of estradiol and also cooperated with Brn-3b to further enhance promoter activity (Figure 6c). These results suggest that stimulation of Brn-3b promoter by ERα can occur independently of estradiol stimulation. We also tested whether increased promoter activation caused by the coexpression of Brn-3b and ERα could also result in enhanced protein expression. For this study, we used the modified BSXE1E construct (Figure 6d), in which the Brn-3b promoter, (containing Brn-3 and ERE elements), drives expression of its own coding sequence. This BSXEIE construct was cotransfected with Brn-3b or ERα expression vectors, alone or together, into MCF-7 cells. Proteins extracted from transfected cells after 48 hours were used for immunoblotting to detect Brn-3b protein. Figure 6e shows that transfected cells coexpressing exogenous Brn-3b and ERα produced higher levels of Brn-3b protein than basal levels in control cells (column 1) or in cells transfected with Brn-3b alone (column 3), where the band represent exogenous as well as endogenous Brn-3b proteins. Thus, coexpression of Brn-3b with ERα at ratios of 1:1 and 1:2 (Brn-3b:ERα) resulted in increased Brn-3b protein, but further increases in ERα (1:4 ratio) resulted in reduced protein levels, which is suggestive of squelching. To demonstrate this squelching effect, we needed to show reduction of Brn-3b protein expression at the higher ratio and this was achieved by reducing exposure times. However, under those conditions, the increases in endogenous Brn-3b following transfection with ERα only were not evident in Figure 6e but can be seen in Figure 6f. Thus, transfecting increasing amounts of ERα expression vector (0.5 -2.0 μg) resulted in increased ERα protein (Figure 6f - top immunoblot), and correlated with enhanced expression of endogenous Brn-3b (lower immunoblot). Therefore, the stimulatory effects of the oestrogen receptor can directly increase transcription from *Brn-3b *gene promoter but also cooperates with Brn-3b to further enhance expression. However this cooperativity is influenced by the ratio of Brn-3b to ERα in cells.

### Mutation of Brn-3 binding sites leads to loss of regulation by ERα

**Figure 7:**
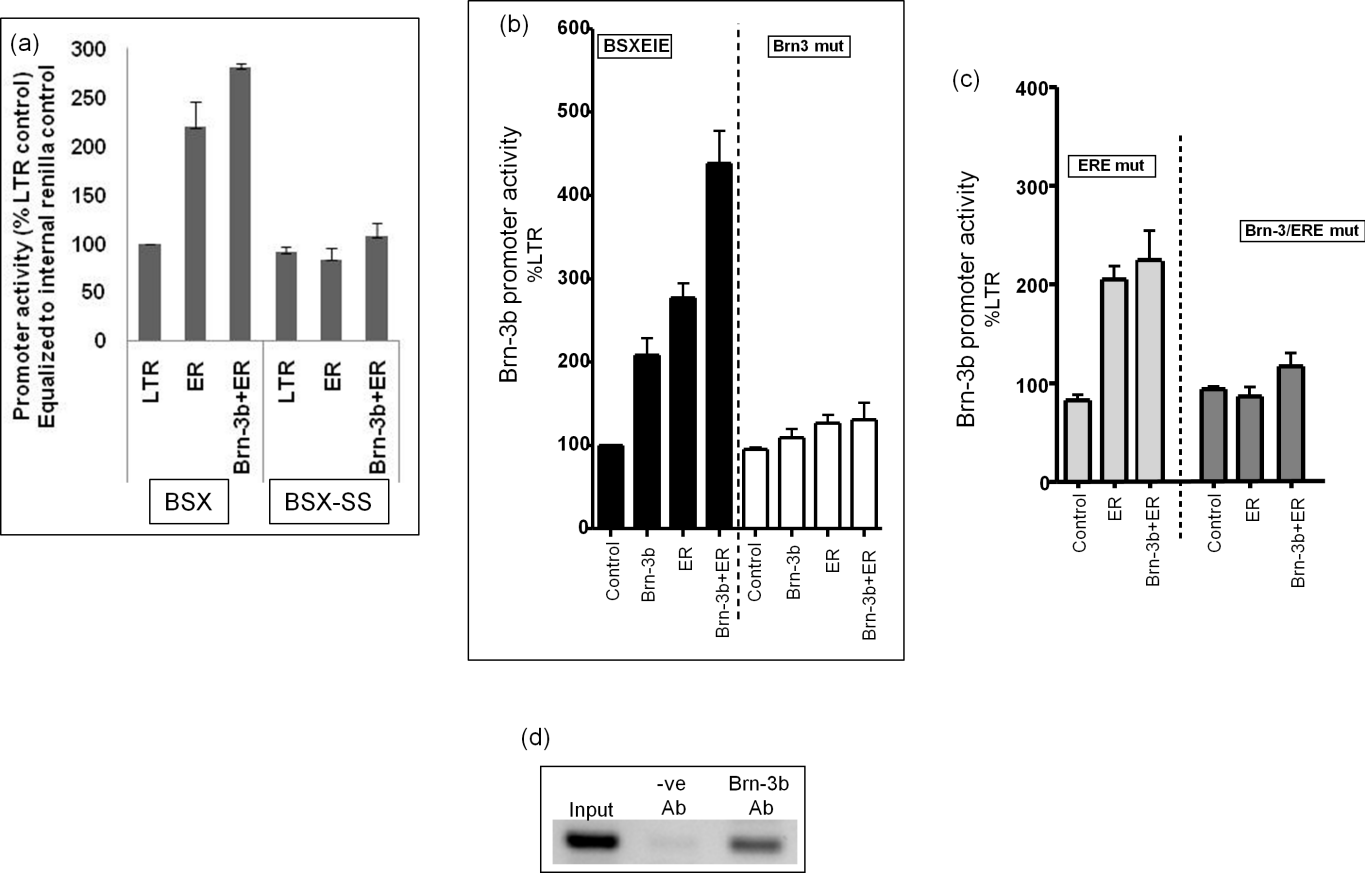
**Mutation of Brn-3 site reduces the inducibility of promoter by ERα and also abolishes cooperativity**. **(a) **Testing effects of ERα with or without Brn-3b on activity of deletion promoter, BS-SS, in which putative binding site for Brn-3b and ERα are lost. Reporter gene activity was adjusted using internal control, Renilla luciferase. Values are shown relative to BS promoter activity in the presence of control empty vector, LTR, set at 100%. Effects of ERα or Brn-3b, alone or together on the intact BS promoter or deletion (BS-SS) promoter are compared **(b) **Reporter gene activity following cotransfection of Brn-3b or ER (alone or together) with WT or mutant Brn-3b reporter construct in which Brn-3b site is mutated. Values have been adjusted for internal Renilla luciferase control and are expressed as percentages of activity in empty vector transfected cells (set at 100%) for the respective promoters. Results represent means ± SD from three independent experiments. **(c) **Similar reporter assays were used to show changes in activity of Brn-3b promoter containing mutation of ERE alone (ERE mutant) or double-mutant lacking both sites (Brn-3/ERE mutant) when coexpressed with Brn-3b +/- ERα. The data represent means ± SD of three independent experiments. **(d) **Representative PCR product obtained using ChIP DNA (immunoprecipitated with Brn-3b antibody) and primers to amplify and promoter region containing the putative Brn-3b binding site. Input column represents PCR amplification using one-tenth of isolated DNA before ChIP. Middle column (-ve Ab) shows product following ChIP assay with negative control α-rabbit Ab only). Right column (Brn-3b Ab) shows product resulting from ChIP assay using Brn-3b Ab.

The BS-SS deletion construct (described in Figure 3b), lacked the Brn-3 and ERE binding sites. Therefore, we analysed the effects of Brn-3b, with or without ERα, on promoter activity and showed loss of inducibility by Brn-3b and ERα (Figure 7a), suggesting that these sites are important for promoter transactivation. We next tested whether these sites were essential for promoter activation, by mutating the Brn-3 consensus sequence (Brn3 mutation in Figure 7b) and ERE (ERE mutation in Figure 7c), either alone or together (Brn3-ERE mutation in Figure 7c), using site-directed mutagenesis. Mutant and WT promoter was then used to test the effects of Brn-3b and ER on promoter on activity following cotransfection studies. Figure 7b (black bars) shows the expected cooperation between Brn-3b and ERα on the WT promoter, whereas mutation of the Brn-3 site (open bars) resulted in loss of induction by Brn-3b but also prevented activation by ERα or cooperative stimulation when ERα is co-expressed with Brn-3b. Mutation of the putative ERE did not affect promoter activity (Figure 7c, light grey bars) but loss of ERE and the adjacent Brn-3 site, in double mutants (Figure 7c, dark grey bars) abolished stimulation by ERα and cooperativity between Brn-3b and ER. These results showing that the stimulatory effects of ERα is not dependent on binding to ERE if the Brn-3b binding site is intact suggest that protein-protein interaction with Brn-3b might facilitate recruitment of ERα to the promoter. Therefore, ER-mediated activation of this promoter is not solely dependent on the ERE site at this position.

Since the Brn-3 site was shown to be important for activation of this promoter, chromatin immunoprecipitation (ChIP) assay was utilised to show that Brn-3b does indeed bind to this site on the promoter *in vivo *in intact cells. Figure 7d shows the PCR product resulting from amplification of promoter sequences containing the Brn-3b site when using Brn-3b ChIP DNA obtained following Chip with Brn-3b antibody from MCF-7 cells overexpressing Brn-3b. PCR primers were used to amplify the promoter region containing the putative Brn-3b site. Input (left column) indicates amplification of chromatin from cells prior to immunoprecipitation, whereas ChIP DNA using Brn-3b Ab (right column) gave rise to significant amplification products, which was not seen following PCR using ChIP DNA with control Ab (middle column). These results therefore confirm that Brn-3b is indeed bound to this region of its own promoter *in vivo *in intact cells.

## Discussion

The mechanisms underlying the development and progression of breast cancer are not fully understood, and this is particularly challenging because of its diverse etiologies [20]. However, it is clear that changes in gene expression are essential to drive different processes that occur during tumourigenesis [21]. Transcription factors control gene expression by binding to specific DNA sequences in gene promoters and often regulate multiple target genes. Because of this ability to control different target genes, deregulation of transcription factors can drive events associated with the initiation and progression of diseases such as cancer [22]. Previous studies have shown that the Brn-3b transcription factor is elevated in >60% of primary breast cancers [1], and when increased, it significantly enhances proliferation and anchorage-independent growth *in vitro *and tumour growth *in vivo *[2,3]. Elevated Brn-3b also confers resistance to growth-inhibitory stimuli and increases the migratory potential of cancer cells [2], suggesting that this transcription factor acts through complex mechanisms in cancer cells. More recent studies have shown increases in Brn-3b in drug-resistant, migratory breast cancer cells (unpublished data, R. Fujita and V. Budhram-Mahadeo). The Brn-3b can give rise to such diverse effects because it regulates different subsets of target genes that control distinct aspects of cellular growth and behavior. For example, Brn-3b might contribute to cellular proliferation by transactivating the promoters of cell cycle regulators, *CDK4 *[4] and *cyclin D1 *[5] whilst repressing the tumour suppressor, *BRCA1 *[6]. However, its effects on drug resistance and migration are likely to be associated with the ability of Brn-3b to regulate other genes, for example, to transactivate *Hsp27 *[7] whilst repressing adhesion molecules, for example, *γ-catenin *[8].

Interestingly, reducing Brn-3b was sufficient to change gene expression and reverse many growth effects [1]. Therefore, Brn-3b can act as a master regulator whose expression profoundly alters the growth of cancer cells. In this regard, Brn-3b might represent an important therapeutic target whose reduction could alter the expression of multiple downstream target genes and thereby reverse their effects on cancer cells. However, to identify strategies for reducing Brn-3b in these cells, we must understand the mechanisms that lead to its increased expression in breast cancer cells.

In this study, we utilised bioinformatics analysis to identify the putative Brn-3b promoter and cloned this regulatory region into a reporter construct for further experimental analysis. By using ChIP assays and site-directed mutagenesis, we identified a key TATA transcriptional start site located at ~278 bp from ATG, which is primarily associated with the expression of *Brn-3b *mRNA in breast cancer cells. Although the upstream initiation site and TA-like elements in the intronic sequence were weakly immunoprecipitated by TBP Ab, these do not appear to be candidates for transcriptional start sites, since the mutation of any or all intronic TA sequences or upstream sequences did not reduce promoter activity, if the start site at -278TATA was intact. This is interesting because an intronic promoter is thought to be important to drive isoform-specific expression of the related *Brn-3a *gene, which has a genomic arrangement similar to that of *Brn-3b*. However, our results suggest that Brn-3b promoter activity in breast cancer cells is driven primarily from the proximal -278TATA site, which is now used to define the transcription start site from this promoter.

Further analysis showed that the Brn-3b promoter can be stimulated by specific growth factors, NGF and EGF, but not by IGF-1, cAMP or TGFβ, and these stimulatory effects require a region of promoter that contains multiple EGFR and SRE sites. The ability of growth factors such as NGF to increase transcription from the Brn-3b promoter is significant because NGF is known to enhance the growth and drive proliferation of breast cancer cells but not of normal breast epithelial cells. Moreover, blocking NGF can inhibit tumour growth and metastasis [16,23], suggesting a key role for NGF in controlling the growth of cancer but not of normal cells. NGF is produced in an autocrine manner by breast cancer cells, and its mitogenic effects in these cells are mediated through the p42/p44 MAPK signalling pathway, since these effects can be blocked by the pharmacological inhibitor PD98059, which targets MEK1 in this pathway [24]. In this study, we showed that stimulation of the Brn-3b promoter by NGF is blocked by PD98059, suggesting that the mitogenic effects of NGF in breast cancer cells may result in part from its ability to increase the expression of regulators such as Brn-3b. The PKC analogue PDBu is also a potent activator of the Brn-3b promoter, and its effects can also be blocked by PD98059, suggesting that this activator converges on the p42/p44 MAPK/ERK1 pathway to stimulate Brn-3b promoter activity. Dominant negative (Dn) MEK also blocked endogenous Brn-3b promoter activity, in a manner that is similar to the ERK1 inhibitor, PD98059. Thus it would appear that the p42/p44 MAPK/ERK pathway is pivotal for activating the Brn-3b promoter and hence expression in breast cancer cells.

In addition to stimulation by growth factors, the Brn-3b promoter is strongly activated by the hormone estradiol, which regulates the growth and proliferation of normal breast epithelium as well as breast cancer cells and is important in the etiology of breast cancer [25]. Oestrogens can regulate gene transcription by acting through one of two receptors: ERα or ERβ. Our results show that overexpression of ERα but not ERβ could strongly stimulate Brn-3b promoter activity. ERα is particularly relevant for the development and progression of breast cancers because it is overexpressed in a significant proportion of breast cancers (> 60%). Furthermore, ER-positive breast cancers are often treated using receptor antagonists, for example, tamoxifen, as a first line of therapy aimed at blocking ER-mediated proliferative effects [26]. Therefore, the ability of ERα to stimulate Brn-3b suggests that the proliferative effects of high ER levels may be associated with the ability of ERα to transactivate other regulators, such as Brn-3b, which in turn can modulate genes associated with growth in these cancer cells either alone or by cooperating with ERα.

The complexity underlying the regulation of the Brn-3b promoter is increased by autoregulation, whereby Brn-3b can weakly stimulate its own expression by binding to recognition sequences present in its promoter. However, cooperation between Brn-3b and ERα could further enhance promoter activity. Such cooperation between Brn-3b and ERα to increase gene expression was previously observed on other ERE-containing target promoters, for example, *HSP27*, where Brn-3b stimulates expression directly by binding to specific sites in the promoter or indirectly by interacting and cooperating with ER to maximally activate this promoter [7]. This ability of Brn-3b to cooperate with ERα to enhance gene expression [9], including its own, is clearly relevant to breast cancer because ER-expressing tumours that are responsive to estradiol will stimulate Brn-3b, which can cooperate with ERα to further increase its own expression. Interestingly, mutation of the putative ERE did not prevent ER-mediated promoter activation when coexpressed with Brn-3b, but mutation of the nearby Brn-3 site abolished activation by ER and its cooperation with Brn-3b. This indicates that ERα could stimulate Brn-3b promoter even if it is not bound to ERE, possibly because interaction with Brn-3b allows recruitment of ER to the promoter. Autoregulation of Brn-3b transcription, either alone or by cooperating with ER, is likely to increase Brn-3b protein expression and subsequently, its target genes in these cells.

Although stimulation of Brn-3b promoter activity by the hormone oestrogen via ERα is likely to act independently and possibly, in parallel with growth factor-mediated promoter activation via the p42/p44 MAPK signalling, there is also significant "cross-talk" between these pathways in breast cancer cells. Thus, estradiol primarily acts through its receptor, ERα, in breast cancer cells, but it can also indirectly stimulate tyrosine kinase receptors, which are also relevant to breast cancer cells. Similarly, transcriptional activity of oestrogen receptor, ERα, is also modulated by p42/p44 MAPK pathway stimulation [27]. Evidence for cross-talk between NGF or EGF and the estradiol pathways has also been demonstrated [28], and in this regard, the anti-oestrogenic drug tamoxifen can inhibit proliferation by EGF or NGF on MCF-7 breast cancer cells [29]. Therefore, diverse pathways, which are stimulated by either hormone or growth factor may act in parallel or converge to stimulate Brn-3b promoter activity and hence increase its expression in breast cancer cells. Evidence for autoregulation by Brn-3b and cooperation with ERα to increase drive its own promoter activity, would suggest that under such circumstances, this feedback loop will maintain high Brn-3b expression. When elevated, Brn-3b is likely to alter the expression of multiple downstream target genes, thereby affecting growth and behaviour in these cancer cells.

## Conclusions

Elevated Brn-3b profoundly enhances tumour growth and confers drug resistance in breast cancer cells, so it is important to identify which factors increase its expression in these cells. In the present studies, we have cloned and analysed the Brn-3b promoter. Furthermore, we have identified key pathways that converge on its promoter to increase activity and hence gene and protein expression in breast cancer cells. Thus, the hormone oestrogen and the growth factors NGF and EGF stimulate the activity of the Brn-3b promoter and subsequently, Brn-3b mRNA and protein expression, suggesting that induction of Brn-3b by such factors will be important in changing the fate of these cells. Increased Brn-3b expression via growth factors such as NGF and EGF or the hormone, estradiol, which are implicated in enhancing the growth of breast cancer cells, are likely to be are propagated by autoregulation. This will lead to changes in multiple Brn-3b target genes which control the growth and behaviour of cancer cells. By elucidating the mechanisms through which regulators such as Brn-3b are increased in cancer cells, we will increase the understanding of how changes are brought about during the development and progression of this disease, and we may also be able to identify strategies to reduce its expression and reverse its effects in breast cancer cells.

## Abbreviations

Brn-3b: transcription factor-related Brn-3a regulator isolated from brain cDNA; BSX-Brn-3b: promoter containing *Bst*X1/*Stu*1/*Xho*1 fragment; ChIP: chromatin immunoprecipitation; EGF: epidermal growth factor; ER: oestrogen receptor; ERα: oestrogen receptor α; ERβ: oestrogen receptor β; ERE: oestrogen response element; GF: growth factor; MAPK: mitogen-activated protein kinase; NGF: nerve growth factor; PI3K: phosphoinositide 3-kinase; POU4F2: member of class 4 subgroup of Pit-Oct-Unc transcription factors; qRT-PCR: quantitative reverse transcriptase polymerase chain reaction; SRE: serum response element; TF: transcription factor; WT: wild type.

## Competing interests

The authors declare that they have no competing interests.

## Authors' contributions

SO mapped transcription start sites, used site-directed mutagenesis and ChIP assays to identify key transcription factor binding sites and helped to identify signalling pathways associated with the regulation of promoter activity. SB, CP and RF were involved in cloning and initial characterization of the promoter. RJH provided reagents required for aspects of the study and invaluable contribution to aspects of these studies. VBM is the principal investigator of the study and was instrumental in designing the study, interpreting and processing the data and preparing the manuscript for publication. All authors read and approved the final manuscript.
